# Multiple insecticide resistance mechanisms in *Anopheles gambiae* s.l. populations from Cameroon, Central Africa

**DOI:** 10.1186/1756-3305-6-41

**Published:** 2013-02-22

**Authors:** Philippe Nwane, Josiane Etang, Mouhamadou Chouaїbou, Jean Claude Toto, Alphonsine Koffi, Rémy Mimpfoundi, Frédéric Simard

**Affiliations:** 1 Organisation de Coordination pour la lutte contre les Endémies en Afrique Centrale, P.O. Box 288, Yaoundé, Cameroon; 2 Université de Yaoundé I, Yaoundé, Cameroon; 3 Faculty of Medicine and Pharmaceutical Sciences, University of Douala, Douala, Cameroon; 4 Institut Pierre Richet (IPR), BP 47, Abidjan, Côte d’Ivoire; 5 MIVEGEC (UMR IRD224-CNRS5290-UM1-UM2), Institut de Recherche pour le Développement (IRD), Montpellier, France

## Abstract

**Background:**

Increasing incidence of DDT and pyrethroid resistance in *Anopheles* mosquitoes is seen as a limiting factor for malaria vector control. The current study aimed at an in-depth characterization of *An. gambiae* s.l. resistance to insecticides in Cameroon, in order to guide malaria vector control interventions.

**Methods:**

*Anopheles gambiae* s.l. mosquitoes were collected as larvae and pupae from six localities spread throughout the four main biogeographical domains of Cameroon and reared to adults in insectaries. Standard WHO insecticide susceptibility tests were carried out with 4% DDT, 0.75% permethrin and 0.05% deltamethrin. Mortality rates and knockdown times (kdt_50_ and kdt_95_) were determined and the effect of pre-exposure to the synergists DEF, DEM and PBO was assessed. Tested mosquitoes were identified to species and molecular forms (M or S) using PCR-RFLP. The hot ligation method was used to depict *kdr* mutations and biochemical assays were conducted to assess detoxifying enzyme activities.

**Results:**

The *An. arabiensis* population from Pitoa was fully susceptible to DDT and permethrin (mortality rates > 98%) and showed reduced susceptibility to deltamethrin. Resistance to DDT was widespread in *An. gambiae* s.s. populations and heterogeneous levels of susceptibility to permethrin and deltamethrin were observed. In many cases, prior exposure to synergists partially restored insecticide knockdown effect and increased mortality rates, suggesting a role of detoxifying enzymes in increasing mosquito survival upon challenge by pyrethroids and, to a lower extent DDT. The distribution of *kdr* alleles suggested a major role of *kdr-*based resistance in the S form of *An. gambiae*. In biochemical tests, all but one mosquito population overexpressed P450 activity, whereas baseline GST activity was low and similar in all field mosquito populations and in the control.

**Conclusion:**

In Cameroon, multiple resistance mechanisms segregate in the S form of *An. gambiae* resulting in heterogeneous resistance profiles, whereas in the M form and *An. arabiensis* insecticide tolerance seems to be essentially mediated by enzyme-based detoxification. Synergists partially restored susceptibility to pyrethroid insecticides, and might help mitigate the impact of vector resistance in the field. However, additional vector control tools are needed to further impact on malaria transmission in such settings.

## Background

Vector borne diseases account for approximately 17% of the estimated global burden of infectious diseases and are the major causes of illness and death in tropical and subtropical countries [1]. In most cases, prevention of these diseases relies on vector control, through the use of insecticide treated materials or indoor residual spraying. Although alternative methods include either vaccine or chemotherapy in certain cases, vector control offers the greatest potential for the large-scale reduction of the disease burden [2]. However, the extensive use of insecticides has led to the development of insecticide resistance, making this strategy less effective and limiting the available options for disease prevention and control [3]. For malaria, vector control is chiefly based on the distribution of long-lasting insecticidal nets (LLINs) and/or indoor spraying of houses with residual insecticides (IRS) [4]. The use of these methods is substantially increasing in endemic countries [5] in the framework of malaria elimination programmes [6]. Evidence of malaria burden reduction through full coverage of LLINs or coupled with IRS are reported in some African countries [7-9].

In Cameroon, apart from a few laboratory and field trials carried out in certain locations [10], IRS is not implemented as a large scale malaria vector control measure. However, the National Malaria Control Programme (NMCP) has been scaling up the use of long-lasting insecticidal nets since 2008, with a free mass distribution of 8,654,731 LLINs branded PermaNet® 2.0 and OlysetNet® throughout the country in 2011. This nationwide distribution of LLINs is undertaken in the context where the main malaria-carrying mosquito vectors, including *An. gambiae* s.s and *An. arabiensis* are reported to exhibit strong resistance to DDT and pyrethroid insecticides [11,12]. This situation is a major concern considering the Roll Back Malaria universal coverage objective by 2015. Malaria vector resistance to insecticides in Cameroon is conferred by two main mechanisms: (1) an increase of detoxification and/or metabolism through high levels of multi-function oxidases (MFOs), glutathione S-transferases (GSTs) and non-specific esterases (NSEs) [12,13] and (2) alterations at site of action in the sodium channel, viz the *kdr* mutations [11,14].

The combined effect of target-site insensitivity and metabolic resistance among malaria mosquito populations remains ambiguous. At the present time, questions over the reliability of single *kdr* genotype in conferring all the variance in resistance phenotype are not unanimously shared in numerous reports [15-18]. Although the impact of *kdr*-based insecticide resistance on the effectiveness of vector control interventions remains to be clearly demonstrated, the knowledge of all operating resistance mechanisms is crucial for success of vector control strategies. Further investigations are therefore needed to assess the co-involvement of *kdr* mutations with other mechanisms in the resistance phenotypes that were previously reported in Cameroon.

In this study, the susceptibility status of *An. gambiae* s.l. populations to DDT and pyrethroid insecticides was assessed after exposure to synergists including PBO (4% pyperonyl butoxide), DEF (0.25% S.S.S-tributyl phosphotritioate) and DEM (8% diethyl maleate) which are known inhibitors of MFOs, NSEs and GSTs, respectively in order to explore the involvement of these detoxifying enzymes in the phenotype of insecticide resistance. Molecular analyses were performed in parallel to assess the association between *kdr* genotypes and resistance phenotype. Biochemical analyses were also performed to assess cytochrome P450 (i.e. MFO) and GST activities in the tested mosquito populations.

## Methods

### Study sites

Mosquitoes were collected from 6 localities in Cameroon chosen on the basis of the selection pressure reported in previous studies [18,19]. These localities belong to the four main biogeographic domains of Cameroon as described in Nwane et al. [14] (Figure ; 1):

**Figure 1:**
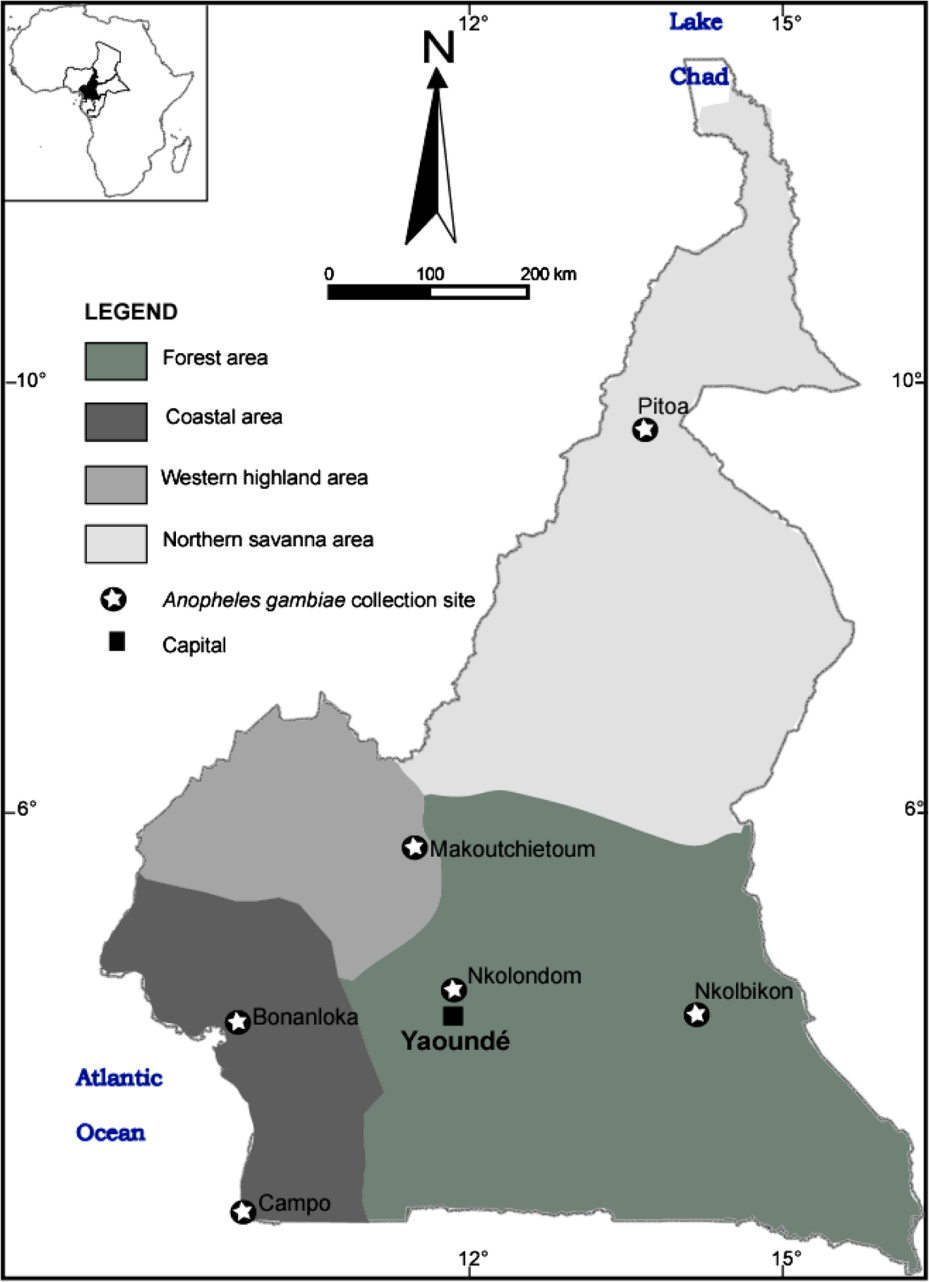
**Map of Cameroon showing *Anopheles gambiae *collection sites.**

1) in the forest area, two collection sites were selected: Nkolondom (03°56’52”N-11°30’18”E) and Nkolbikon (05°36’06”N-13°40’30”E). The former is a market gardening area located in the outskirts of Yaoundé (the capital city of Cameroon) and the latter is a suburban area of Bertoua city located in the eastern part of the country;

2) in the coastal area, two collection sites were selected: Bonanloka (04°01’43”N-09°43’54”E), an urban area of Douala, the economic capital of Cameroon, and Campo (02°22’30”N-09°49’33”E), a rural zone situated southwest of Douala in the coastal region and characterized by intensive forest exploitation and timber storage;

3) in the highland area, samples were collected in Makoutchietoum (05°36’37”N-10°36’24”E), a locality with extensive and manual gardening;

4) in the northern savanna area, samples were collected in Pitoa (09°23’31”N-13°30’09”E), a locality surrounded by cotton fields and situated at about 15 km from Garoua in the northern region of the country.

### Mosquito collections

*An. gambiae* s.l. larvae and pupae were collected between October 2008 and May 2009. In each collection site, *c.a.* 30 breeding sites were prospected and larvae were collected and reared locally until adult emergence. Adult mosquitoes were sexed and identified using morphological identification reference keys [20,21]. Only female *An. gambiae* s.l. were used for bioassays, as well as molecular and biochemical analyses. The Kisumu susceptible strain of *An. gambiae* was used as a reference strain to compare the susceptibility level of the field collected samples as well as the activity levels of the tested detoxifying enzymes.

### Insecticide susceptibility bioassays

Bioassays were performed on mosquitoes aged 2–4 days using WHO susceptibility test kits and standard protocol for adults [3] under ambient room temperature ranging from 25°C to 28°C and relative humidity of 70-80%. Impregnated filter papers with 4% DDT, 0.05% deltamethrin and 0.75% permethrin were supplied by the Vector Control and Research Unit, University Sains Malaysia (Penang, Malaysia). Each full set of bioassays was performed with five batches of 20–25 unfed females: four batches were exposed to insecticide-impregnated filter papers and one batch was exposed to untreated filter paper and served as a control. Tests were concomitantly performed with the Kisumu susceptible reference strain of *An. gambiae* maintained in OCEAC (Yaoundé, Cameroon) insectaries. The number of mosquitoes knocked down was recorded at 5 minute intervals during the 1 h-long exposure and mortality was determined 24 h post exposure. After completion of the mortality counts, dead and surviving mosquitoes were separately kept on desiccant (silica gel) and stored at −20°C for molecular analyses. Unexposed (control) mosquitoes were also individually kept in 0.5 ml Eppendorf tubes and stored at −80°C for biochemical analyses, together with a batch of unexposed Kisumu specimens.

### Synergist bioassays

Synergist bioassays were performed on adult female mosquitoes using 3 synergists namely 0.25% S.S.S-tributyl phosphotritioate (DEF, ChemServices West Chester, PA), an inhibitor of esterases, 8% diethyl maleate (DEM, Sigma Milwaukee, WI), an inhibitor of GSTs and 4% pyperonyl butoxide (PBO, Sigma Milwaukee, WI), an inhibitor of oxidases. The preparation of stock solutions for each synergist and impregnation on filter papers (12 cm × 15 cm) were performed in the “Laboratoire de Recherche sur le Paludisme de l’OCEAC (Yaoundé, Cameroon)”. For each test run, two treatments were compared: the insecticide alone versus a combination of synergist and insecticide. It is noteworthy highlighting that during the assay including synergist and insecticide, mosquitoes were first exposed for 1 h to a filter paper impregnated with synergist followed by 1 h exposure to the insecticide.

### Species, molecular forms identification and *kdr* genotyping

DNA was extracted from each selected specimen using the method of Collins et al. [22] and each individual was identified to the species level and molecular form using PCR-RFLP [23]. This method allows simultaneous identification of the M and S molecular forms within *An. gambiae* s.s., as well as the other species of the *An. gambiae* complex. Alleles at the *kdr* locus were genotyped using hot oligonucleotide ligation assay (HOLA) as described by Lynd et al. [24].

### Biochemical assays

In this study, only mixed function oxidases (MFOs) and glutathione S-transferase (GSTs) activity were evaluated in female mosquitoes aged 2–4 days reared from larvae and never exposed to insecticides but used as control while performing susceptibility tests. Forty-seven specimens were assayed per microtitre plate according to the method described by Hemingway [25].

### Statistical analysis

The knockdown times for 50% and 95% of tested mosquitoes (kdt_50_ and kdt_95_) were estimated using a log-time probit model [26]. Mortality rates were compared between bioassays performed with insecticide alone and after pre-exposure to synergists using Mantel-Haenszel chi-square tests. The rate of suppression of knockdown effect by synergists (kds) was computed as described by Thomas et al. [27] with effective values above 10%. Biochemical assay data (enzymatic activity per mg of protein) of wild specimens of *An. gambiae* s.l were compared to the Kisumu susceptible strain using Wilcoxon rank sum test and all computations were performed using R software (Version 2.15.2, R Development Core Team 2005).

## Results

### Resistance phenotypes and the effect of pre-exposure to synergists

A total of 80 susceptibility tests were performed, including 12 tests with mosquitoes of the reference susceptible strain Kisumu (e.g., 3 tests with DDT, permethrin or deltamethrin alone and 9 combinations whereby mosquitoes were first exposed to the synergist DEF, DEM or PBO then to the insecticide at the diagnostic dose) and 68 tests with wild samples. Because of low sample sizes in Nkolbikon, some synergist-insecticide combinations could not be performed: DDT was only tested alone and after initial exposure to DEM and mosquitoes exposed to DEM were not tested for susceptibility to the pyrethroids permethrin and deltamethrin.

Throughout the assays, the Kisumu strain of *An. gambiae* displayed mortality rates above 99% for the 3 insecticides, with no impact of pre-exposure to synergists (Figure ; 2). The kdt_50_ values were 19.1, 9.5 and 8.8 minutes for DDT, deltamethrin and permethrin, respectively; the corresponding kdt_95_ values were 29.8, 17.3 and 14.6 minutes, respectively and the rates of knockdown time suppression (kdts) were less than 10% in all cases (not shown).

**Figure 2:**
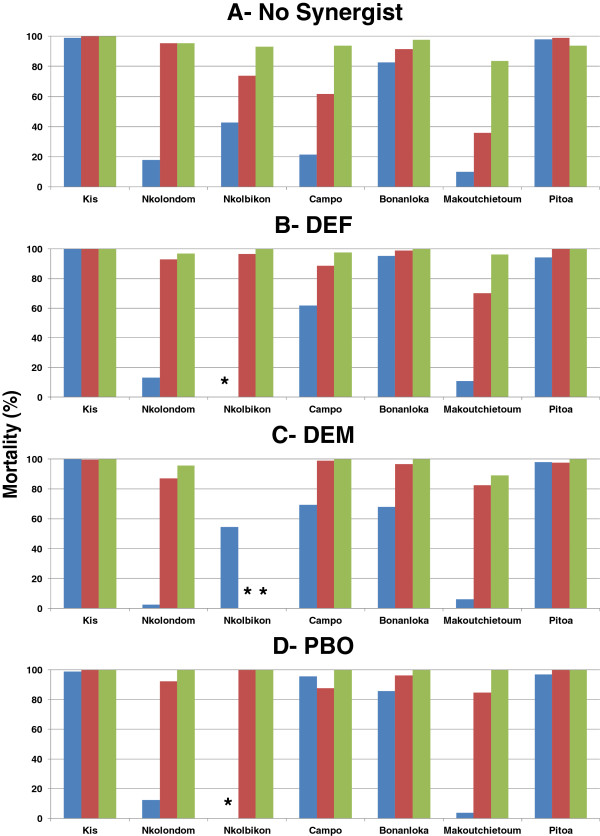
**Mortality rates in *Anopheles gambiae *24-hours post exposure to 4% DDT (Blue), 0.75% permethrin (Red) and 0.05% deltamethrin (Green) with and without pre-exposure to synergists.** (**A**): No pre-exposure to synergists; (**B**): Pre-exposure to DEF; (**C**): Pre-exposure to DEM; (**D**): Pre-exposure to PBO. * Not determined because the assay was not performed.

Field mosquito populations displayed variable levels of resistance to the three insecticides tested (Figure ; 2A). High mortality to DDT, permethrin and deltamethrin was observed in the Pitoa sample (mortality rate > 93%) and, to a lower extent, in the Bonanloka sample (mortality rate > 82%) with low, if any, effect of pre-exposure to synergists on both mortality rates (Figure ; 2) and kdt_50_ values (Table 1). In contrast, the mosquito population from Makoutchietoum was highly resistant to DDT and permethrin with mortality rates below 40%, and showed reduced susceptibility to deltamethrin. Pre-exposure to synergists significantly increased mortality to the two pyrethroid insecticides but did not affect resistance to DDT in this population (Figure ; 2). The kdt_50_ values for DDT were above 60 min with and without synergists, whereas pre-exposure to DEF, DEM and PBO led to a significant decrease in kdt_50_ values for both pyrethroid insecticides (Table 1). A similar pattern was observed in Nkolbikon and Campo populations, although mortality to DDT also increased significantly after pre-exposure to synergists in the latter, and especially so with PBO (Figure ; 2A and D). Finally in Nkolondom, mortality rates to permethrin and deltamethrin were above 95% in all treatments, whereas mortality to DDT was dramatically low and remained below 20% whether or not tested mosquitoes were previously exposed to any of the synergists used in this study.

**Table 1:** **Knockdown times and percent suppression of knockdown recorded in six*Anopheles gambiae.*populations from Cameroon**

**Locality**	**Test**	**N**	**kdT**_**50**_**[CI**_**95**_**] (min)**	**kdT**_**95**_**[CI**_**95**_**] (min)**	**%kdts**
Nkolondom	4%DDT	92	>60	>60	
	0.25%DEF + 4%DDT	89	>60	>60	61.7
	8%DEM + 4%DDT	86	>60	>60	-
	4%PBO + 4%DDT	82	>60	>60	< 0
	0.05%Delta	89	20.1 [17.1-22.8]	45.0 [39.0-58.3]	
	0.25%DEF + 0.05%Delta	94	23.1 [22.0-24.1]	42.8 [40.4-45.9]	< 0
	8%DEM + 0.05%Delta	89	21.4 [20.3-22.3]	37.8 [35.5-40.8]	< 0
	4%PBO + 0.05%Delta	89	20.9 [20.0-21.9]	36.6 [34.5-39.3]	< 0
	0.75%Perm	83	40.2 [37.5-42.8]	>60	
	0.25%DEF + 0.75%Perm	87	38.1 [36.3-40.0]	>60	5.1
	8%DEM + 0.75%Perm	85	40.0 [38.1-42.1]	>60	0.3
	4%PBO + 0.75%Perm	90	33.8 [32.3-45.3]	>60	15.8
Nkolbikon	4%DDT	91	>60	>60	
	8%DEM + 4%DDT	86	>60	>60	< 0
	0.05%Delta	87	17.1 [16.1-18.1]	33.7 [31.4-36.7]	
	0.25%DEF + 0.05%Delta	62	21.1 [19.7-22.4]	43.6 [40.1-48.4]	< 0
	4% PBO + 0.05%Delta	87	14.1 [12.0-16.1]	42.1 [36.0-52.0]	17.5
	0.75%Perm	88	33.7 [30.4-36.9]	>60	
	0.25%DEF + 0.75%Perm	89	20.9 [18.9-22.9]	>60	37.9
	4%PBO + 0.75%Perm	87	13.7 [12.1-21.2]	>60	59.3
Campo	4%DDT	87	>60	>60	
	0.25%DEF + 4%DDT	89	50.1 [44.8-58.1]	>60	51.8
	8%DEM + 4%DDT	85	43.8 [40.5-47.9]	>60	57.9
	4%PBO + 4%DDT	87	43.7 [41.1-46.7]	>60	58.0
	0.05%Delta	95	13.7 [12.7-14.7]	34.1 [31.3-37.4]	
	0.25%DEF + 0.05%Delta	88	14.5 [13.6-15.4]	29.9 [27.8-32.8]	<0
	8%DEM + 0.05%Delta	86	17.9 [16.9-18.2]	29.7 [27.9-32.1]	<0
	4%PBO + 0.05%Delta	86	20.1 [19.1-21.1]	36.4 [34.2-39.2]	<0
	0.75%Perm	88	37.3 [34.1-40.6]	>60	
	0.25%DEF + 0.75%Perm	80	22.7 [19.2-25.8]	>60	39.1
	8%DEM + 0.75%Perm	87	47.3 [38.8-59.6]	>60	<0
	4%PBO + 0.75%Perm	82	15.5 [14.1-16.8]	41.0 [37.3-45.9]	58.4
Bonanloka	4%DDT	87	37.9 [36.5-39.5]	>60	
	0.25%DEF + 4%DDT	83	33.2 [31.8-34.6]	>60	12.4
	8% DEM + 4%DDT	81	48.6 [46.4-51.2]	>60	<0
	4%PBO + 4%DDT	84	37.9 [34.6-41.9]	>60	0
	0.05%Delta	78	12.8 [10.7-14.7]	24.1 [20.4-31.6]	
	0.25%DEF +0.05%Delta	83	15.3 [12.3-17.9]	31.4 [26.0-42.9]	<0
	8%DEM + 0.05%Delta	84	18.9 [18.0-19.8]	28.5 [26.9-30.7]	<0
	4%PBO + 0.05%Delta	88	14.3 [11.5-16.8]	29.5 [24.4-40.6]	<0
	0.75%Perm	81	15.2 [13.2-17.1]	35.3 [30.7-42.6]	
	0.25%DEF + 0.75%Perm	92	19.5 [18.6-20.4]	33.9 [31.9-36.5]	<0
	8%DEM + 0.75%Perm	89	16.1 [14.6-17.6]	30.9 [27.8-35.7]	<0
	4% PBO + 0.75%Perm	80	12.4 [11.2-13.4]	23.8 [21.9-26.5]	18.4
Makoutchietoum	4%DDT	90	>60	>60	
	0.25%DEF + 4%DDT	81	>60	>60	27.7
	8%DEM + 4%DDT	80	>60	>60	-
	4%PBO + 4%DDT	82	>60	>60	21.2
	0.05%Delta	86	35.3 [33.9-36.7]	>60	
	0.25%DEF +0.05%Delta	83	22.2 [21.2-23.1]	34.3 [32.6-36.7]	37.1
	8%DEM + 0.05%Delta	83	30.1 [28.9-31.3]	51.8 [48.9-55.6]	14.7
	4%PBO + 0.05%Delta	88	30.3 [28.9-31.6]	>60	14.2
	0.75%Perm	84	>60	>60	
	0.25%DEF + 0.75%Perm	89	>60	>60	33.7
	8%DEM + 0.75%Perm	86	>60	>60	36.5
	4%PBO + 0.75%Perm	78	48.7 [46.1-51.9]	>60	50.2
Pitoa	4%DDT	100	39.3 [37.9-40.6]	>60	
	0.25%DEF + 4%DDT	91	38.1 [36.9-39.3]	58.9 [56.1-62.7]	3.1
	8%DEM + 4%DDT	87	36.7 [35.5-37.9]	58.9 [55.9-62.7]	6.6
	4%PBO + 4%DDT	100	37.4 [33.3-41.2]	>60	4.8
	0.05%Delta	96	10.5 [8.8-12.1]	21.2 [17.9-27.3]	
	0.25%DEF + 0.05%Delta	97	7.2 [6.6-7.7]	13.3 [12.1-15.0]	31.4
	8%DEM + 0.05%Delta	94	9.0 [8.3-9.7]	19.2 [17.6-21.4]	14.3
	4%PBO + 0.05%Delta	100	18.6 [17.5-19.8]	45.4 [41.9-49.7]	<0
	0.75%Perm	81	16.8 [12.5-20.2]	39.7 [32.7-55.2]	
	0.25%DEF + 0.75%Perm	91	11.2 [9.9-13.6]	32.1 [27.4-39.6]	33.3
	8%DEM + 0.75%Perm	84	18.5 [17.5-19.4]	26.8 [25.3-28.9]	< 0
	4%PBO + 0.75%Perm	94	13.1 [12.5-13.6]	19.3 [18.1-20.9]	22.1

*Delta*: deltamethrin; *Perm*: permethrin; *PBO*: pyperonyl butoxide; *DEF*: S.S.S-tributyl phosphotritioate; *DEM*: diethyl maleate; *N*: sample size; kdT_50_ and kdT_95_: knockdown times for 50% and 95% of the tested population; %kdts: percent of knockdown time suppression; CI95: confidence interval at 95%; min: minute.

### Mosquito diversity and *kdr* allelic frequency distribution in insecticide-resistant mosquitoes

Molecular analyses were performed on 721 specimens that were randomly sampled from among survivors to DDT (N = 417), permethrin (N = 244) and deltamethrin (N = 60).

Table 2A shows the distribution of species and molecular forms within *An. gambiae* s.l. mosquitoes that survived exposure to DDT. In all localities, the S form of *An. gambiae* was amongst the survivors. In Nkolondom, Nkolbikon and Makoutchietoum, it was the only one member of the complex found amongst DDT survivors, with a high frequency of *kdr* 1014F resistant allele (f > 0.77), presence of the *kdr* 1014S resistant allele (0.02 < f < 0.23) and low frequency of the 1014L susceptible allele (f < 0.11). A similar pattern was observed in the S sample from Campo. However, the 1014L susceptible *kdr* allele was predominant in the few S form specimens that survived DDT exposure in Bonanloka and Pitoa (f > 0.63), as well as in survivors of the M form detected in Bonanloka and Campo (f > 0.58) and *An. arabiensis* from Pitoa (f = 1).

**Table 2:** **Species diversity and allelic frequencies at the*kdr*locus in survivor mosquitoes exposed to insecticides**

**A- DDT**		**N**	**%**	**f(1014L)**	**f(1014F)**	**f(1014S)**
Nkolondom	***An. gambiae*S**	**96**	**100**	**0.03**	**0.95**	**0.02**
	*An. gambiae* M	0	0	-	-	-
	*An. arabiensis*	0	0	-	-	-
	*An. gambiae* S	73	100	0.11	**0.78**	0.11
Nkolbikon	*An. gambiae* M	0	0	-	-	-
	*An. arabiensis*	0	0	-	-	-
	*An. gambiae* S	6	33	**0.67**	0.25	0.08
Bonanloka	*An. gambiae* M	12	67	**0.58**	0.38	0.04
	*An. arabiensis*	0	0	-	-	-
	*An. gambiae* S	68	85	0.00	**0.79**	0.21
Campo	*An. gambiae* M	12	15	**0.67**	0.33	0.00
	*An. arabiensis*	0	0	-	-	-
	*An. gambiae* S	135	100	0.00	**0.77**	0.23
Makoutchietoum	*An. gambiae* M	0	0	-	-	-
	*An. arabiensis*	0	0	-	-	-
	*An. gambiae* S	4	27	**0.63**	0.38	0.00
Pitoa	*An. gambiae* M	0	0	-	-	-
	*An. arabiensis*	11	73	**1.00**	0.00	0.00
**B- Permethrin**	N	%	f(1014L)	f(1014F)	f(1014S)	
	*An. gambiae* S	27	100	0.00	**0.98**	0.02
Nkolondom	*An. gambiae* M	0	0	-	-	-
	*An. arabiensis*	0	0	-	-	-
	*An. gambiae* S	20	100	0.00	**1.00**	0.00
Nkolbikon	*An. gambiae* M	0	0	-	-	-
	*An. arabiensis*	0	0	-	-	-
	*An. gambiae* S	11	22	**0.82**	0.18	0.00
Bonanloka	*An. gambiae* M	39	78	**0.64**	0.36	0.00
	*An. arabiensis*	0	0	-	-	-
	*An. gambiae* S	52	98	0.00	**0.84**	0.16
Campo	*An. gambiae* M	1	2	**1.00**	0.00	0.00
	*An. arabiensis*	0	0	-	-	-
	*An. gambiae* S	91	100	0.00	**0.95**	0.05
Makoutchietoum	*An. gambiae* M	0	0	-	-	-
	*An. arabiensis*	0	0	-	-	-
	*An. gambiae* S	0	0	-	-	-
Pitoa	*An. gambiae* M	0	0	-	-	-
	*An. arabiensis*	3	100	**1.00**	0.00	0.00
**C- Deltamethrin**	N	%	f(1014L)	f(1014F)	f(1014S)	
	*An. gambiae* S	14	100	0.00	**0.96**	0.04
Nkolondom	*An. gambiae* M	0	0	-	-	-
	*An. arabiensis*	0	0	-	-	-
	*An. gambiae* S	5	100	0.00	**1.00**	0.00
Nkolbikon	*An. gambiae* M	0	0	-	-	-
	*An. arabiensis*	0	0	-	-	-
	*An. gambiae* S	0	0	-	-	-
Bonanloka	*An. gambiae* M	2	100	**1.00**	0.00	0.00
	*An. arabiensis*	0	0	-	-	-
	*An. gambiae* S	8	100	0.00	**0.81**	0.19
Campo	*An. gambiae* M	0	0	-	-	-
	*An. arabiensis*	0	0	-	-	-
	*An. gambiae* S	26	100	0.00	**0.88**	0.12
Makoutchietoum	*An. gambiae* M	0	0	-	-	-
	*An. arabiensis*	0	0	-	-	-
	*An. gambiae* S	0	0	-	-	-
Pitoa	*An. gambiae* M	0	0	-	-	-
	*An. arabiensis*	5	100	**1.00**	0.00	0.00

A) 4% DDT, B) 0.75% permethrin and C) 0.05% deltamethrin.

Table 2B and 2C shows a similar pattern for permethrin and deltamethrin survivors, respectively. As for DDT, the S form of *An. gambiae* was widespread amongst permethrin survivors and was found in most study sites. Frequency of the *kdr* resistant alleles was high in these groups (f (1014F) > 0.81) with no occurrence of the 1014L susceptible allele, except in the S form sample from Bonanloka. Again, the 1014L allele was predominant, and often the only one *kdr* allele found in M form specimens from Bonanloka and Campo, as well as in *An. arabiensis* specimens from Pitoa that survived exposure to pyrethroids.

In summary, the S form of *An. gambiae* was widespread and was shown to survive DDT and pyrethroid exposure in most sampled localities. Survival was associated with high frequencies of *kdr* resistant alleles, especially allele 1014F. In Bonanloka however, the susceptible 1014L allele was found in high frequency in DDT and permethrin survivors and no S form specimen was identified in deltamethrin survivors. M form and *An. arabiensis* specimens were also identified amongst DDT and permethrin survivors in two and one localities, respectively, with no indication of any correlation with resistant alleles’ frequencies at the *kdr* locus.

### Genotype at the *kdr* locus and resistance to permethrin

To further explore the relationship between genotype at the *kdr* locus and resistance phenotype, we compared the distribution of genotypic frequencies at the *kdr* locus in permethrin resistant (i.e. ‘survivors’, N = 244) and susceptible (i.e. ‘dead’, N = 219) mosquitoes. Figure ; 3A shows high genotypic diversity in the S form samples, with all possible genotypes being represented in most ‘dead’ samples, whereas paired ‘survivors’ samples were significantly enriched in 1014F homozygotes (p < 0.05). This supports a role of *kdr* mutations in shaping resistance to permethrin in these mosquito populations. In the Bonanloka sample however, genotypic diversity was much lower, with only two genotypes identified (i.e. 1014L/1014L and 1014L/1014F, Figure ; 3A) and no difference in genotypic frequencies between dead and survivors. Low genetic and genotypic diversity was also observed within the M form samples from Bonanloka and Campo (Figure ; 3B) with limited differences in the distribution of genotypic frequencies between dead and survivor groups suggesting limited, if any, role of the *kdr* mutations in resistance to permethrin in these populations. Furthermore, all *An. arabiensis* mosquitoes analyzed in this study (N = 56) were from Pitoa and were homozygous for the 1014L susceptible allele.

**Figure 3:**
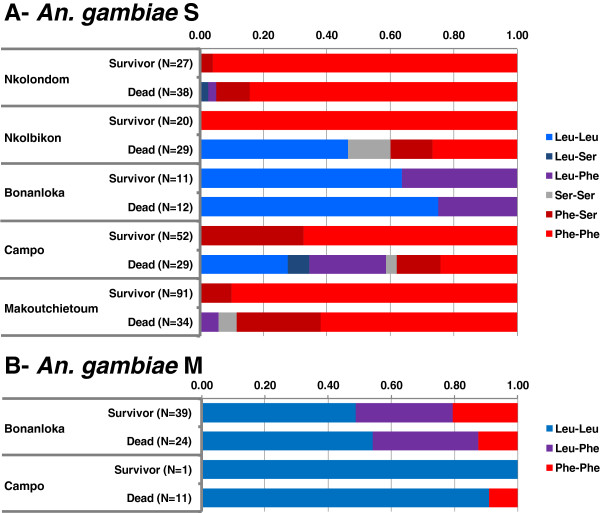
**Frequency distribution of the different genotypes at position 1014 of the *kdr *locus in samples of dead and survivor mosquitoes after exposure to 0.75% permethrin.A**) *An. gambiae* S form; **B**) *An. gambiae* M form. Colour key to the different genotypes is given in the insert on the right. Leu: Leucine at position 1014 (encoded by allele 1014L); Phe: Phenylalanine at position 1014 (encoded by allele 1014F); Ser: Serine at position 1014 (encoded by allele 1014S).

### Mixed function oxidases (MFOs) and glutathione S-transferases (GSTs) activity

Biochemical assays were successfully performed on samples from 5 out of the 6 studied *An. gambiae* s.l. populations. Figure ; 4A shows the mean level of MFOs activity (expressed in cytochrome P450 units) in field-collected mosquitoes compared to the reference susceptible strain Kisumu. Except in Nkolondom, all sampled populations showed a significantly higher MFOs activity than the susceptible reference strain Kisumu (p < 0.05). In contrast, the level of GST activity in these populations was not significantly different (p > 0.05) from that of the Kisumu strain (Figure ; 4B).

**Figure 4:**
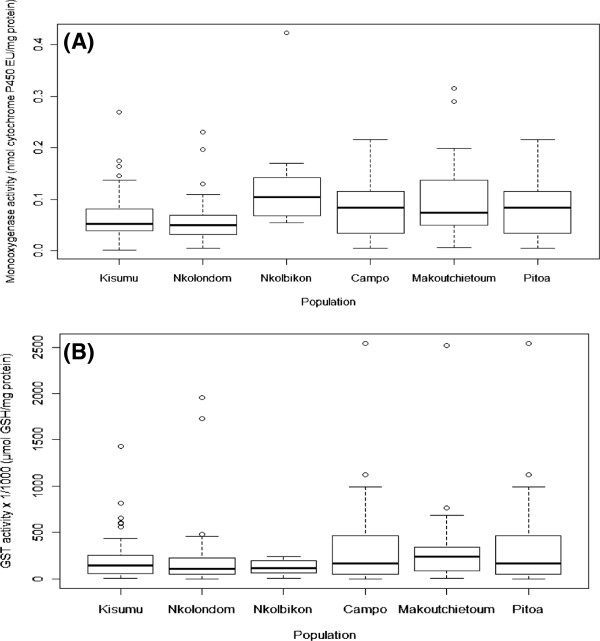
**Mean level of detoxifying enzyme activity in field-collected *Anopheles gambiae *s.l. from Cameroon.** (**A**) Mixed function oxidases (MFOs); (**B**) Glutathion S-transferases (GSTs). Kisumu refers to the reference susceptible strain of *An. gambiae* s.s used as control.

## Discussion

This study highlighted the diversity of insecticide resistance phenotypes in *Anopheles gambiae* s.l. populations from Cameroon. The *An. arabiensis* population sampled in Pitoa was fully susceptible to DDT and permethrin and showed reduced susceptibility to deltamethrin, as previously reported from this area [12,19]. On the other hand, resistance to DDT was widespread in *An. gambiae* s.s. populations and heterogeneous levels of susceptibility to pyrethroid insecticides permethrin and deltamethrin that rarely reached full susceptibility were observed. This is also consistent with previous reports [14,18,28]. In many cases, exposing mosquitoes to synergists (i.e. DEF, DEM and PBO, respectively) prior to insecticides partially restored insecticide knockdown effect and increased mortality rates in standard WHO assays, suggesting a role of detoxifying enzymes (i.e. NSEs, GSTs and MFOs, respectively) in increasing mosquito survival upon challenge by permethrin, deltamethrin and, to a lower extent, DDT. Molecular and biochemical investigations further revealed a complex interplay between molecular (i.e., *kdr-*based) and metabolic (i.e., enzyme-based) resistance mechanisms in mosquitoes surviving insecticide exposure. Table 3 shows a qualitative summary of the different putative resistance mechanisms that were evidenced in this study, based on i) synergist bioassays results, ii) detection of *kdr* resistant alleles and iii) biochemical assessment of detoxifying enzyme activities (e.g., NSEs and MFOs). Multiple resistance mechanisms segregated in the S form of *An. gambiae* resulting in heterogeneous resistance profiles, whereas in the M form and *An. arabiensis* insecticide tolerance seems to be essentially mediated by enzyme-based detoxification.

**Table 3:** **Putative insecticide resistance mechanisms identified in different*An. gambiae*s.l. populations from Cameroon**

**Species/Population**	**Resistance mechanism**				
	**Detoxifying Enzymes**		***Kdr*Alleles**		
	**MFOs**	**GSTs**		**1014F**	**1014S**
***An. gambiae*S form**					
Nkolondom	**-**	**-**		**++**	**+**
Nkolbikon	**+**	**-**		**++**	**+**
Campo	**+**	**-**		**++**	**+**
Bonanloka	**N/A**	**N/A**		**+**	**+**
Makoutchietoum	**+**	**-**		**+**	**+**
Pitoa	**+**	**-**		**+**	**-**
***An. gambiae*M form**					
Campo	**++**	**-**		**+**	**-**
Bonanloka	**N/A**	**N/A**		**+**	**-**
***An. arabiensis***					
Pitoa	**+**	**-**		**-**	**-**

++: Major role in resistance; +: Presence; -: Not observed in this study; N/A: Not determined.

Several approaches may be used to investigate mechanisms of insecticide resistance in a vector population. So far, cross-resistance to commonly used classes of insecticides based on bioassay data has been suggested to depict resistance mechanisms [29], but this approach does not provide irrefutable evidence when metabolic resistance and target-site insensitivity interact in a particular population. The entry point of an investigation on multiple mechanisms is the co-formulation of synergists with the insecticide to counteract metabolic resistance. Synergists act by blocking metabolic pathways that would otherwise break down insecticides, then restore the susceptibility to the insecticide [30-32]. Using synergists in the current study allowed gaining preliminary information on metabolic resistance mechanisms co-involved with *kdr* mutations in *An. gambiae* s.l. resistance in Cameroon.

Complete or partial DDT and permethrin resistance suppression was achieved in the presence of the three synergists in the Campo sample, suggesting a major role for metabolic processes in shaping the resistance phenotype of this mosquito population encompassing both *An. gambiae* S and M molecular forms. In other populations such as in Nkolondom and Makoutchietoum where only the S form survived insecticide exposure, the resistance level to DDT was not affected by synergists, suggesting no role for metabolic resistance mechanisms and a major effect for *kdr* alleles in shaping resistance to DDT in these populations [18,28]. Pre-exposure to synergists, however, restored susceptibility to pyrethroids in Makoutchietoum, reflecting an impact of metabolic processes in pyrethroid resistance, whereas high initial mortality to both permethrin and deltamethrin in Nkolondom suggested absence of metabolic detoxification in this latter population.

The synergistic effects of PBO were noticed with all three insecticides (deltamethrin, permethrin and DDT to some extent). These observations are consistent with previous reports on the mode of action and synergist efficacy of PBO [33-36] and agree with a predominant position of PBO in synergizing a wide range of insecticides including organophosphates, carbamates, pyrethrins and pyrethroids [37,38]. Furthermore, high level activity of cytochrome P450s (i.e., MFOs) was detected in most of the *An. gambiae* s.l. populations surveyed, confirming their involvement in the phenotype of resistance as revealed by bioassay tests with synergists. These data complement previous reports on metabolic based insecticide resistance in *An. gambiae* s.s. from Cameroon [12,13]. Overexpression of P450s enzymes has been found to play a major role in pyrethroid resistance in insects [39-41]; likewise, high level GSTs activity was reported to be associated with insect resistance to DDT and pyrethroids [42-44]. Both bioassay and biochemical data presented in this study are congruent with the first observation but not with the second. The absence of correlation between low levels of GST activity and DDT resistance may be due to the involvement of *kdr* mutations as the major DDT resistance mechanism in tested mosquito populations. Because it is known that PBO inhibits P450s that mediate resistance to all classes of insecticides [45,46] and to the well known organochlorine DDT [47,48], our findings suggest that, when this synergist is associated with deltamethrin, it could be efficiently used for malaria vector control interventions as reported in recent studies [49,50]. The potent possibility of PBO as an effective synergist for deltamethrin has also been reported against *Aedes* and *Culex* genera, suggesting its wide range of action in several mosquito species [36]. Even though the activity of NSEs was not assessed in the framework of this study, bioassay data revealed that DEF was also an effective synergist to suppress pyrethroid resistance in at least some of the *An. gambiae* s.l. populations sampled. Moreover, in previous studies, NSEs were shown to be inhibited by PBO [51-53] and it is therefore possible that NSEs further contribute to insecticide resistance in Cameroon. However, it is clear that the effects of DEF, DEM and PBO as shown in the current study did not reveal the specificity between each synergist and a given enzyme family. Pasay et al. [32] concluded that, the metabolic routes blocked by synergists are not yet fully understood and may be dependent on the species of arthropods. Hence, further investigations are needed to evaluate the level of involvement of each enzyme family in the overall metabolic-based resistance observed in *An. gambiae* s.l. populations from Cameroon.

## Conclusion

The current study revealed the simultaneous presence of multiple resistance mechanisms in the malaria vector *An. gambiae* s.l. populations from Cameroon, a pattern that likely holds true for most parts of West and Central Africa [54-57]. The co-occurrence and co-implication of both metabolic- and *kdr-*based resistance mechanisms in *An. gambiae* s.l is a serious threat to the effectiveness of current malaria vector control operations based on LLINs and IRS. Because malaria is a devastating disease with considerable impact on human health in Cameroon and beyond [58] urgency might require the use of synergists to mitigate insecticide resistance in major malaria vector mosquitoes. However, alternative innovative vector control tools and solutions are urgently needed to complement or even replace insecticide-based strategies in order to face the challenge of global malaria elimination [5,59].

## Competing interests

The authors declare that they have no competing interests.

## Authors’ contributions

JE and FS conceive the study. JE, FS, RM, and PN designed the study protocol; JE, PN, MC and JCT, performed field work and bioassays; PN and AK have performed molecular and biochemical analyses; PN and JE analyzed and interpreted the data; PN drafted the manuscript which was critically revised by JE, RM and FS. All the authors read and approved the final manuscript.

## References

[B1] WHO Global strategic framework for integrated vector management 2004 Geneva, Switzerland: WHO

[B2] Townson H Nathan MB Zaim M Guillet P Manga L Bos R Kindhauser M Exploiting the potential of vector control for disease prevention Bull World Health Organ 2005 83 942 947 16462987 PMC2626501

[B3] WHO Test procedures for insecticide resistance monitoring in malaria vectors, bio-efficacy and persistence of insecticides in treated surfaces WHO/CDS/CPC/MAL/98.12 1998 Geneva, Switzerland: WHO, Control of Communicable Diseases (CDS) Prevention and Control

[B4] WHO Indoor residual spraying: use of indoor residual spraying for scaling up malaria control and elimination 2008 Geneva, Switzerland: World Health Organization

[B5] Feachem R Sabot O A new global malaria eradication strategy Lancet 2008 371 1633 1635 10.1016/S0140-6736(08)60424-9 18374409

[B6] WHO World malaria report 2009 2009 Geneva: World Health Organization 40

[B7] Overgaard HJ Reddy VP Abaga S Matias A Reddy MR Kulkarni V Schwabe C Segura L Kleinschmidt I Slotman MA Malaria transmission after five years of vector control on Bioko island, Equatorial Guinea Parasites Vectors 2012 5 253 10.1186/1756-3305-5-253 23146423 PMC3533880

[B8] Bhattarai A Ali AS Kachur P Martensson A Abbas AK Khatib R Almafazy A Ramsan M Rotllant G Gerstenmaier JF Molteni F Abdulla S Montgomery SM Kaneko A Bjorkman A Impact of artemesinin-based combination therapy and insecticide-treated nets on malaria burden in Zanzibar PloS Med 2007 4 e309 10.1371/journal.pmed.0040309 17988171 PMC2062481

[B9] Okumu FO Moore SJ Combining indoor residual spraying and insecticide-treated nets for malaria control in Africa: overview of possible outcomes and an outline of suggestions for the future Malaria J 2011 10 208 10.1186/1475-2875-10-208 PMC3155911 21798053

[B10] Etang J Nwane P Mbida JA Piameu M Manga B Souop D Awono-Ambene P Variations of insecticide residual bio-efficacy on different types of walls: results from a community-based trial in south Cameroon Malaria J 2011 10 333 10.1186/1475-2875-10-333 PMC3233609 22047173

[B11] Etang J Fondjo E Chandre F Brengues C Nwane P Chouaϊbou M Ndjemai H Simard F First report of *knockdown* mutations in the malaria vector *anopheles gambiae* from Cameroon AmJTrop Med Hyg 2006 74 795 797 16687682

[B12] Mϋller P Chouaїbou M Pignatelli P Etang J Walker ED Donnelly MJ Simard F Ranson H Pyrethroid tolerance is associated with elevated expression of antioxidants and agricultural practice in *anopheles arabiensis* sampled from an area of cotton fields in northern Cameroon Mol Ecol 2008 17 4 1145 1155 18179425 10.1111/j.1365-294X.2007.03617.x

[B13] Etang J Manga L Toto JC Guillet P Fondjo E Chandre F Spectrum of metabolic-based resistance to DDT and pyrethroids in *anopheles gambiae* s.l populations from Cameroon J Vect Ecol 2007 32 1 123 133 10.3376/1081-1710(2007)32[123:SOMRTD]2.0.CO;2 17633433

[B14] Nwane P Etang J Chouaїbou M Toto JC Mimpfoundi R Simard F *Kdr-*based insecticide resistance in *anopheles gambiae* s.s populations in Cameroon: spread of the L1014F and L1014S mutations BMC Res Notes 2011 4 463 10.1186/1756-0500-4-463 22035176 PMC3221647

[B15] Brooke BD *Kdr:* can a single mutation produce an entire insecticide resistance phenotype? Trans Roy Soc Trop Med Hyg 2008 102 524 525 10.1016/j.trstmh.2008.01.001 18295809

[B16] Donnelly MJ Corbel V Weetman D Wilding CS Williamson MS Black WC IV Does *kdr* genotype predict insecticide-resistance phenotype in mosquitoes Trends Parasitol 2009 25 5 213 219 10.1016/j.pt.2009.02.007 19369117

[B17] Ramphul U Boase T Bass C Okedi LM Donnelly MJ Mϋller P Insecticide resistance and its association with target-site mutations in natural populations of *anopheles gambiae* from eastern Uganda Trans Roy Soc Trop Med Hyg 2009 103 1121 1126 10.1016/j.trstmh.2009.02.014 19303125

[B18] Nwane P Etang J Chouaibou M Toto JC Kerah-Hinzoumbé C Mimpfoundi R Awono-Ambene HP Simard F Trends in DDT and pyrethroid resistance in *anopheles gambiae* s.s. Populations from urban and agro-industrial settings in southern Cameroon BMC Infect Dis 2009 9 163 10.1186/1471-2334-9-163 19793389 PMC2764715

[B19] Chouaїbou M Etang J Brevault T Nwane P Hinzoumbé CK Mimpfoundi R Simard F Dynamics of insecticide resistance in the malaria vector *anopheles gambiae* s.l from an area of extensive cotton cultivation in northern Cameroon Trop Med Int Health 2008 13 4 1 11 18248566 10.1111/j.1365-3156.2008.02025.x

[B20] Gillies MT De Meillon B The anophelinae of Africa south of the Sahara Publ South African Institute Med Res 1968 54 343p

[B21] Gillies MT Coetzee M Supplement to the anophelinae of Africa south of the Sahara (afrotropical region) South African Institute Med Res 1987 55 143

[B22] Collins FH Mendez MA Razmussen MO Mehaffey PC Besansky NJ Finnerty VA Ribosomal RNA gene probe differentiates member species of *Anopheles gambiae* complex AmJTrop Med Hyg 1987 37 37 41 10.4269/ajtmh.1987.37.37 2886070

[B23] Fanello C Santolamazza F Ddella Torré A Simultaneous identification of species and molecular forms of the *Anopheles gambiae* complex by PCR-RFLP Med Vet Entomol 2002 16 461 464 10.1046/j.1365-2915.2002.00393.x 12510902

[B24] Lynd A Ranson H McCall PJ Randle NP Black WC IV Walker ED Donnelly MJ A simplified high-throughput method for pyrethroid knockdown resistance (*kdr*) detection in *anopheles gambiae* Malaria J 2005 4 16 10.1186/1475-2875-4-16 PMC555548 15766386

[B25] Hemingway J Techniques to detect insecticide resistance mechanisms (Field and laboratory manual). Document WHO/CDS/CPC/MAL/98.6 1998 Geneva, Switzerland: World Health Organ

[B26] Finney DJ Probit analysis 1971 3 Cambridge, UK: Cambridge University Press 333

[B27] Thomas A Kumar S Pillai MMK Pyperonyl butoxide as a counter measure for deltamethrin resistance in *Culex quinquefasciatus* Say Entomon 1991 18 1 10

[B28] Antonio-Nkondjio C Tene Fossog B Ndo C Menze Djantio B Zebaze Togouet S Awono-Ambene P Costantini C Wondji C Ranson H *Anopheles gambiae* distribution and insecticide resistance in the cities of douala and Yaoundé (Cameroon): influence of urban agriculture and pollution Malaria J 2011 10 154 10.1186/1475-2875-10-154 PMC3118161 21651761

[B29] Brodgon WG McAllister JC Vulule J Heme peroxydase activity measured in single mosquitoes identifies individuals expressing an elevated oxidase for insecticide resistance J Am Mosq Control Assoc 1997 13 233 237 9383763

[B30] Casida JE Mixed-function oxydases involvement in the biochemistry J Agric Food Chem 1970 18 753 772 4919838 10.1021/jf60171a013

[B31] Jao LT Casida JE Insect pyrethroid-hydrolyzing esterases Pestic Biochem Physiol 1974 4 465 472 10.1016/0048-3575(74)90071-6

[B32] Pasay C Arlian L Morgan M Gunning R Rossiter L Holt D Walton S Beckman S McCarthy J The effect of insecticide synergists on the response of scabies mites to pyrethroid acaricides PLoS Negl Trop Dis 2009 3 e354 10.1371/journal.pntd.0000354 19125173 PMC2603020

[B33] Farnham AW Jones DG The mode of action of piperonyl butoxide with reference to studying pesticide resistance Piperonyl butoxide: the insecticide synergist 1998 London: Academic 199 214

[B34] Pap L Arvai G Bertok B Kuruczne RZ Bakonyvari I Comparative of new synergists containing a butynyl-type synergophore group and piperonyl butoxide derivatives Pest Manag Sci 2001 57 186 190 10.1002/1526-4998(200102)57:2<186::AID-PS290>3.0.CO;2-Z 11455649

[B35] Fakoorziba MR Eghbal F Vijayan VA Synergist efficacy of piperonyl butoxide with deltamethrin as pyrethroid insecticide on *Culex tritaeniorhyncus* (diptera: culicidae) and other mosquito species Environ Toxicol 2008 24 19 24 18442073 10.1002/tox.20386

[B36] Cakir G Yavuz O Kokak O Effects of piperonyl butoxide and tetramethrin combinations on biological activities of selected synthetic pyrethroid insecticides against different housefly (*musca domestica* L., diptera: muscidae) populations Acta Veterinaria Brno 2008 77 464 474

[B37] Tomlin CSD The pesticide manual 1997 11 Farnham: British Crop Protection Council 1606

[B38] Kaene P Jones DG The use of piperonyl butoxide in formulations for the control of pests of humans, domestic and food animals Piperonyl butoxide: the insecticide synergist 1998 London: Academic Press London 289 300

[B39] Feyereisen R Insect P450 enzymes Annu Rev Entomol 1999 44 507 533 10.1146/annurev.ento.44.1.507 9990722

[B40] Kasai S Scott JD Over expression of cytochrome P450 CYP6D1 is associated with monooxygenases-mediated pyrethroid resistance in house flies from Georgia Pestic Biochem Physiol 2000 68 34 41 10.1006/pest.2000.2492

[B41] Hemingway J Hawkes NJ McCarroll L Ranson H The molecular basis of insecticide resistance in mosquitoes Insect Biochem Mol Biol 2004 34 653 666 10.1016/j.ibmb.2004.03.018 15242706

[B42] Vontas JG Small GJ Hemingway J Glutathione S-transferase as anti oxidant defence agents confer pyrethroids in *Nilaparvata lugens* Biochem J 2001 357 65 72 10.1042/0264-6021:3570065 11415437 PMC1221929

[B43] Grant DF Dietze EC Hammock BD Glutathione S-transferase isozymes in *aedes aegypti* purification, characterization and isozyme-specific regulation Insect Biochem 1991 21 421 433 10.1016/0020-1790(91)90009-4

[B44] Zhou ZH Syvanen M A complex glutathione transferase gene family in the housefly *musca domestica* Mol Gen Genet 1997 256 187 194 10.1007/s004380050560 9349710

[B45] Hodgson E Levi PE Jones DG Interactions of piperonyl butoxide with cytochrome P450 Piperonyl butoxide: the insecticide synergist 1998 London: Academic Press 41 53

[B46] Kumar S Thomas A Sahgal A Verma A Samuel T Pillai MK Effect of the synergist, piperonyl butoxide on the development of resistance in yellow fever mosquito, *Aedes aegypti* L, (Diptera: Culicidae) Arch Insect Biochem Physiol 2002 50 1 8 10.1002/arch.10021 11948970

[B47] Chung H Bogwitz MR McCart C Andrianopoulos A French-Constant RH Batterham P Daborn PJ Cis-regulatory elements in the accord retrotransposon result in tissue-specific expression of the *drosophila melanogaster* insecticide resistance gene Cyp6g1 Genetics 2007 175 1071 1077 17179088 10.1534/genetics.106.066597 PMC1840086

[B48] Chiu TL Wen Z Rupasinghe SG Schuler MA Comparative molecular modelling of *anopheles gambiae* CYP6Z1, a mosquito P450 capable of metabolizing DDT Proc Natl Acad Sci 2008 105 8855 8860 10.1073/pnas.0709249105 18577597 PMC2449330

[B49] Corbel V Chabi J Dabiré RK Etang J Nwane P Pigeon O Akogbéto M Hougard JM Field efficacy of a new mosaic long lasting mosquito net (PermaNet® 3.0 Against pyrethroid-resistant malaria vectors: a multi centre study in western and central Africa Malaria J 2010 9 113 10.1186/1475-2875-9-113 PMC2877060 20423479

[B50] Yewhalaw D Asale A Tushune K Getachew Y Duchateau L Speybroeck L Bio-efficacy of selected long-lasting insecticidal nets against pyrethroid resistant *anopheles arabiensis* from south-western Ethiopia Parasites Vectors 2012 5 159 10.1186/1756-3305-5-159 22871143 PMC3485103

[B51] Gunning RB Moores GD Devonshire AL Jones DG Inhibition of resistance-related esterases by piperonyl butoxide in *helicoverpa armigera* (Lepidoptera: noctuidae) and *aphis gossypi* (hemiptera: aphididae, pp. 215–226 Piperonyl butoxide 1998 San Diego, CA: Academic

[B52] Young SJ Gunning RV Moores GD The effect of pyperonyl butoxide on pyrethroid-resistance associated esterases in *helicoverpa armigera* (hubner) (Lepidoptera; noctuidae) Pest Manag Sci 2005 61 397 401 10.1002/ps.996 15605351

[B53] Khot AC Bingham G Field LM Moores GD A novel assay reveals the blockade of esterases by piperonyl butoxide Pest Manag Sci 2008 64 1139 1142 10.1002/ps.1603 18481337

[B54] Kerah-Hinzoumbé C Péka M Nwane P Donan-Gouni I Etang J Samè-Ekobo A Simard F Insecticide resistance in *Anopheles gambiae* from southwestern Chad, Central Africa Malaria J 2008 7 192 10.1186/1475-2875-7-192 PMC2566574 18823537

[B55] Djègbè I Olayidé B Aboubakar S Thibaud M Ranson H Chandre F Akogbéto M Corbel V Dynamics of insecticide resistance in malaria vectors in Benin: first evidence of the presence of L1014S kdr mutation in *anopheles gambiae* from west Africa Malaria J 2011 10 261 10.1186/1475-2875-10-261 PMC3179749 21910856

[B56] Namountougou M Simard F Baldet T Diabaté A Ouédraogo JB Martin T Dabiré RK Multiple insecticide resistance in *anopheles gambiae* s.l. Populations from Burkina Faso, west Africa PLoS One 2012 7 11 e48412 10.1371/journal.pone.0048412 23189131 PMC3506617

[B57] Basilua Kanza JP El Fahime E Alaoui S Essassi M Brooke B Nkebolo Malafu A Watsenga Tezzo F Pyrethroid, DDT and malathion resistance in the malaria vector *anopheles gambiae* from the democratic republic of Congo Trans Roy Soc Trop Med Hyg 2013 107 1 8 14 23222943 10.1093/trstmh/trs002

[B58] WHO World malaria report 2012 2012 Geneva: World Health Organization 195

[B59] Takken W Knols BGJ Malaria vector control: current and future strategies Trends Parasitol 2009 25 3 101 104 10.1016/j.pt.2008.12.002 19168392

